# Serotonergic agents and linezolid: Impact of exposure to more than one agent concomitantly on risk of adverse effects

**DOI:** 10.1017/ash.2023.260

**Published:** 2023-09-29

**Authors:** Xuping Yan, Christopher McCoy, Ryan Chapin, Matthew Lee, Howard Gold, Kendall Donohoe

## Abstract

**Background:** The off-target effects linezolid have the potential to cause serotonin syndrome when given in conjunction with serotonergic agents. Despite package insert labeling as a contraindication, several postmarketing studies have demonstrated a low incidence of serotonin syndrome with the concomitant use of linezolid and other serotonergic agents. Linezolid provides a convenient oral option for gram-positive infections. However, due to concerns for serotonin syndrome, the use of linezolid is sometimes avoided. **Methods:** We performed a single-center, retrospective, medical record review of all adult inpatients from September 2021 to September 2022. Patients included had 1 administration of linezolid and 1 inpatient administration of a selective serotonin reuptake inhibitor (SSRI) or serotonin and norepinephrine reuptake inhibitor (SNRI) within 14 days. The primary outcome was the incidence of serotonin syndrome as defined by the Hunter serotonin toxicity criteria, which were retrospectively applied to each patient based on medical-record documentation. We compared patients receiving 1 versus multiple serotonergic agents. Secondary outcomes included duration of hospitalization and adverse outcomes based on concerns for serotonin syndrome such as need for rescue, ICU admission, or change in medication. **Results:** Of the 50 included patients from a convenience sample, 27 (54%) were on linezolid and >1 serotonergic agent. Patients had similar baseline characteristics (Table 1). The most common concomitant agent used was an SSRI. Other agents that predispose patients to serotonin syndrome included opioid analgesics and other classes of antidepressants (Fig. 1). Serotonin syndrome occurred within 48 hours in 1 patient on an SNRI and a continuous fentanyl drip. There was no need for rescue or ICU admission due to serotonin syndrome. No patients were readmitted due to serotonin syndrome, and no differences were observed in hospital lengths of stay. **Conclusions:** Exposure to a single serotonergic agent combined with receipt of linezolid was not associated with any cases of serotonin syndrome. Exposure to multiple serotonergic agents was not associated with a high incidence of serotonin syndrome. This small series supports previous reports demonstrating relative safety of linezolid given with serotonergic agents and encourages review of interruptive drug–drug interaction alerts for linezolid within the electronic ordering system.

**Figure:**
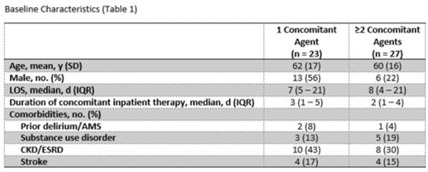

**Figure:**
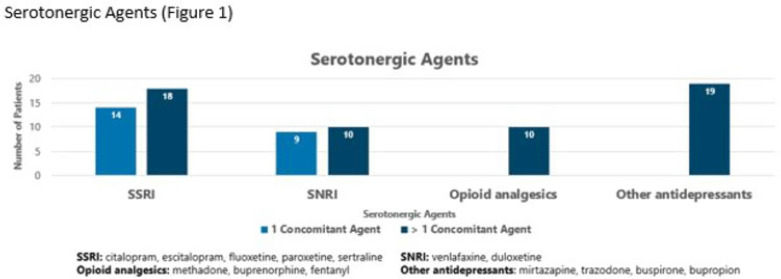

**Disclosures:** None

